# Comprehensive metabolomics combined with machine learning for the identification of SARS-CoV-2 and other viruses directly from upper respiratory samples

**DOI:** 10.1128/jcm.02042-24

**Published:** 2025-10-09

**Authors:** Catherine A. Hogan, Anthony T. Le, Afraz Khan, LingHui David Su, ChunHong Huang, Malaya K. Sahoo, Chieh-Wen Lo, Marwah Karim, Karin Ann Stein, Shirit Einav, Tina M. Cowan, Benjamin A. Pinsky

**Affiliations:** 1 British Columbia Centre for Disease Control Public Health Laboratory, Vancouver, British Columbia, Canada; 2 University of British Columbia 8166, Vancouver, British Columbia, Canada; 3 Biochemical Genetics Laboratory, Stanford Health Care, Palo Alto, California, USA; 4 Department of Pathology, Stanford University School of Medicine 10624 , Stanford, California, USA; 5 Clinical Virology Laboratory, Stanford Health Care, Palo Alto, California, USA; 6 Department of Medicine, Division of Infectious Diseases and Geographic Medicine, Stanford University 196258, Stanford, California, USA; 7 Chem-H, Stanford University 553601, Stanford, California, USA; 8 Department of Microbiology and Immunology, Stanford University 316781, Stanford, California, USA; 9 Chan Zuckerberg Biohub 578083, San Francisco, California, USA; St Jude Children's Research Hospital, Memphis, Tennessee, USA

**Keywords:** metabolomics, nasopharyngeal, SARS-CoV-2, COVID-19, variants of concern, machine learning, respiratory viruses

## Abstract

Metabolic profiling of respiratory samples from individuals infected and uninfected with respiratory viral infections may identify biomarker signatures that complement routine clinical diagnostic testing and offer unique insights into pathophysiology. We used liquid chromatography quadrupole time-of-flight mass spectrometry to generate untargeted metabolomic profiles and identified top biomarker signatures differentiating severe acute respiratory syndrome coronavirus type 2 (SARS-CoV-2) positive from negative samples via machine learning. We then adapted these signatures to liquid chromatography-tandem mass spectrometry for targeted profiling and assessed classification performance, including samples positive for other respiratory viruses and negative for viral testing. A total of 1,226 samples were tested, including 521 positive samples for SARS-CoV-2, 97 for influenza A, 96 for respiratory syncytial virus (RSV), 211 for other respiratory viruses, and 301 negative samples. The top-performing model was the Light Gradient Boosting Model, which showed an area under the receiver operating characteristic curve (AUC) of 0.99 (95% confidence interval [CI], 0.99–1.00), sensitivity of 0.96 (95% CI, 0.91–0.99), and specificity of 0.95 (95% CI, 0.90–0.97). A separate machine learning analysis investigating the performance by viral subtype showed high performance for the identification of influenza A virus with an AUC of 0.97 (95% CI, 0.94–0.99) and RSV with an AUC of 0.99 (95% CI, 0.97–1.00). The two features with the highest ranking were identified as 3-oxo-heneicosanoic acid and 2-(4-hydroxyphenyl) ethanol. These findings extend our understanding of the metabolic impact of respiratory viral infections and support the potential of metabolomics to complement routine clinical diagnostic methods.

**IMPORTANCE:**

Molecular testing has greatly improved how viruses are diagnosed; however, gaps remain, including limited sensitivity directly from specimens and inability to differentiate active from resolved infection. In this study, we investigated the use of a distinct diagnostic approach, mass spectrometry for detection of metabolites (small molecules) combined with machine learning analysis, for the diagnosis of SARS-CoV-2 and other respiratory viruses. We demonstrated strong performance of this approach directly from upper respiratory swab samples to differentiate SARS-CoV-2-infected versus uninfected individuals. Extension of this approach to influenza and RSV maintained a high level of performance. This research suggests that mass spectrometry-based infectious disease diagnostic testing has clinical potential and that these metabolomic features may reveal novel host-pathogen interactions and therapeutic targets. Applying a similar approach to prospective, multisite cohorts of patients with other infectious diseases carries potential to extend our understanding of the metabolic pathways involved in the host response to infection.

## INTRODUCTION

The clinical presentations of respiratory viral infections overlap substantially, and accurate diagnostic testing is required to identify the causal etiological agent for better clinical management. This is particularly important in an era of increased accessibility of targeted antiviral therapy, including for influenza and coronavirus disease 2019 (COVID-19) (1, 2). Respiratory viruses have been associated with distinct metabolomic profiles that may differentiate infected from uninfected individuals directly from respiratory samples, serum, and plasma (3–5). Direct testing of upper respiratory tract samples is of clinical interest as it is less invasive than phlebotomy, aligns with current routine testing practice for respiratory virus infections, and may closely correlate with metabolomic changes at the site of viral infection as investigated previously for influenza (3).

For severe acute respiratory syndrome coronavirus type 2 (SARS-CoV-2), a consistent trend across studies has been the finding of reduced circulating serotonin levels in acute infection, including as a marker of clinical severity (6, 7) and in individuals with long COVID-19 (8), likely driven by several mechanisms, including upregulation of interferon genes. These studies were performed during a period where SARS-CoV-2 dominated the epidemiology of respiratory viruses and largely in the absence of circulating influenza virus. Now that SARS-CoV-2 is part of the landscape of routine respiratory viruses, there is a need to investigate the performance of metabolomics for the identification of SARS-CoV-2 in the context of other circulating respiratory viruses.

In this study, we used liquid chromatography/quadrupole time-of-flight (LC/Q-TOF) on upper respiratory samples combined with machine learning analysis to identify a top biomarker feature signature as a potential discriminator between SARS-CoV-2-infected and uninfected individuals. This signature was then adapted to simpler and more sensitive testing by targeted liquid chromatography-tandem mass spectrometry (LC/MS-MS, referred to as tandem mass spectrometry) in an independent validation cohort. We subsequently combined virus culture with tandem mass spectrometry for metabolic profiling of virally infected and uninfected cells. Finally, we pursued chemical synthesis of the top feature and performed tandem mass spectrometry testing to further confirm identification.

## MATERIALS AND METHODS

### Study population and sample collection

We identified individuals infected with SARS-CoV-2, influenza A, respiratory syncytial virus (RSV), human metapneumovirus (hMPV), adenovirus (AdV), rhinovirus (RV), and parainfluenza viruses (PIVs) 1–4 from respiratory swab samples (including nasal, mid-turbinate and nasopharyngeal swabs) collected in viral transport medium at Stanford Health Care, Stanford Tri-Valley, Stanford Children’s Health and affiliated clinics and outpatient centers in the San Francisco Bay Area. All samples were selected through convenience sampling, whereby available samples with sufficient residual volume were included. Samples from the biomarker signature discovery cohort were collected from symptomatic inpatient and outpatient individuals between 22 December 2021 and 14 January 2022. Negative samples in the discovery cohort were tested only for SARS-CoV-2 by nucleic acid amplification testing (NAAT). Samples for the validation cohort were collected from symptomatic inpatient and outpatient individuals between 23 December 2021 and 31 May 2023. Negative samples in the validation cohort were collected from symptomatic inpatient and outpatient individuals who tested negative for SARS-CoV-2, influenza A, influenza B, and RSV by NAAT. All samples were stored at –80°C until thawed for processing for metabolomics testing, and no formal freeze-thaw or sample stability testing was performed. SARS-CoV-2 NAAT was performed using one of several emergency-use authorized commercial SARS-CoV-2 tests, including the Aptima SARS-CoV-2 and Panther Fusion SARS-CoV-2 Assays (both from Hologic Inc., Marlborough, MA, USA), Xpert Xpress SARS-CoV-2, SARS-CoV-2 plus, SARS-CoV-2/Flu/RSV, and SARS-CoV-2/Flu/RSV plus (all from Cepheid, Sunnyvale, CA, USA), SARS-CoV-2 reverse transcription quantitative PCR (RT-qPCR) Reagent Kit (PerkinElmer, Shelton, CT, USA), New Coronavirus Nucleic Acid Detection Kit (Revvity, Waltham, MA, USA), and the ePlex Respiratory Pathogen Panel 2 (GenMark Diagnostics, Carlsbad, CA, USA). Genotyping was performed by RT-qPCR as part of laboratory variant surveillance, and SARS-CoV-2 whole-genome sequencing was performed on a subset of these samples, as previously described (9). NAAT for the other respiratory viruses was carried out using the Food and Drug Administration-cleared Hologic Panther Fusion FluA/B/RSV, AdV/hMPV/RV, and Paraflu Assays (Hologic, Inc.).

### Metabolic profiling by LC/Q-TOF on the discovery cohort

Metabolic profiling of samples was performed by LC/Q-TOF using an in-line two-column reversed-phase and ion-exchange method, as previously described (10). In brief, a volume of 50 µL of VTM was mixed with 200 µL of isopropanol and centrifuged at 4°C for 15 min at 17,000 × *g*. Chromatographic separation was performed using a series of two columns: column 1, an HSS T3, 1.8 µm, 2.1 mm ID × 50 mm (Waters Corporation, Milford, MA, USA); column 2, Intrada Amino Acid, 3 µm, 2 mm ID × 30 mm (Imtakt, Portland, OR, USA), on a Waters class H quaternary pump (Waters Corporation). Three eluants were used: eluant A: 4 mL formic acid, 0.1 mL ammonium hydroxide in 1 L water; eluant B: 2.7 mL formic acid, 0.74 mL ammonium hydroxide in 1 L methanol; and eluant C: 10 mL formic acid, 11 mL ammonium hydroxide in 1 L water. Detection was performed on a Waters Cyclic Ion Mobility Separation Quadrupole Time of Flight Mass Spectrometer (CIMS Q-TOF) with electrospray ionization (ESI). An injection volume of 5 µL was used with a runtime of 20 min. Samples were analyzed in positive ion mode in a randomized manner using a mass range of *m*/*z* 50–1,200 with the CIMS Q-TOF in V-mode, with a lock mass of leucine enkephalin (m + H/z = 556.2771 and m − H/z = 554.2615). Peak picking, peak alignment, and qualitative analysis were performed using Progenesis QI v.1.0.0.1 (Waters Corporation) using D5-pyroglutamate as an internal standard. The resulting table of retention time, accurate mass, peak area, and peak height was then directly exported from Progenesis QI to Microsoft Excel.

### Fragmentation

Fragmentation experiments of the leading 20 features were performed using a pooled sample consistent with all samples run in the LC/Q-TOF on the discovery cohort. The same LC/Q-TOF method as above was used but with a collision energy of 10, 20, and 30 V at the transfer cell of the Q-TOF. The 20 V collision energy produced the best results and was retained for the subsequent phase of the study. This collision energy is equivalent to a Q2 collision cell on a typical quadrupole time-of-flight mass spectrometer. Multiple fragments were identified for each biomarker using the known retention time and molecular ion of the desired biomarkers.

### Targeted testing by LC/MS-MS on the validation cohort

Using the Q1 molecular ion and identified Q3 fragment ions, the two-column method was repeated using tandem mass spectrometry. After confirmation of the Q1/Q3 selective reaction monitoring (SRM), a modified analytical method was used to reduce analysis time. Chromatographic separation was performed using a WARP 2.7 µm 90A C18 tapered ID × 50 mm column (Premier LCMS) on a Class H quaternary pump (Waters Corporation) for the full cohort. Two eluants were used: eluant A: 0.1% formic acid water; eluant B: 0.1% formic acid methanol. The flow rate was at 0.45 mL/min with a gradient formation of initial conditions of 99% A, 1% B; at 0.3 min, 99% A, 1% B; at 1.5 min, 82% A, 18% B; at 1.7 min, 82% A, 18% B; and at 2 min, 99% A, 1% B. Detection was performed on a Xevo TQ-XS Triple Quadrupole Mass Spectrometer with ESI (Waters Corporation). An injection volume of 5 µL was used with a runtime of 3.5 min. Samples were analyzed in positive ion mode.

### Identification of the top differentiating SARS-CoV-2 biomarkers

To determine the chemical structures of the top differentiating compounds, the mass-to-charge ratios (m/z) of the intact and fragmented metabolites were used to query the METLIN Gen2 database (https://metlin.scripps.edu/). Candidate biomarkers were then confirmed by obtaining the commercially available compounds, 2-(4-hydroxyphenyl) ethanol and 4-ethoxyphenol (Sigma-Aldrich, St. Louis, MO, USA), or, if not commercially available, synthesizing the compound internally (3-oxo-heneicosanoic acid, synthesis protocol). We pursued this approach with purchased or synthesized compounds to confirm the biomarker signature and enable the most robust tier 1 match of the top feature of interest. Biomarker identification by mass spectrometry was performed using the same method as for the targeted testing by LC/MS-MS described above, with the exception of using an orthogonal column for confirmation. Chromatographic separation was performed using a WARP 2 2.7 µm 90A C18 tapered ID × 50 mm column (Premier LCMS) on a class H quaternary pump (Waters Corporation). The SRM pairs and retention times of these standards were compared to those observed in clinical samples.

### Virus culture

Once the biomarker signature was identified through untargeted and targeted metabolomics as above, we pursued virus culture combined with tandem mass spectrometry of the supernatant from SARS-CoV-2-infected and SARS-CoV-2-uninfected cells. A549-ACE2 (BEI Resources, NR-53821) and Vero E6-TMPRSS2 (JCRB Cell Bank, #JCRB1819) were maintained in Dulbecco’s modified Eagle medium (DMEM) supplemented with 10% fetal bovine serum (FBS), 1% penicillin-streptomycin, and 1 mg/mL G418 (all cell culture reagents from Gibco Scientific, Waltham, MA, USA). All cell lines were maintained in a humidified incubator at 37°C with 5% CO_2_ and tested negative for *Mycoplasma* by MycoAlert (Lonza, Morristown, NJ, USA). SARS-CoV-2/wild-type (USA-WA1/2020) viral stock was generated in Vero E6-TMPRSS2 cells, as previously described (11). Whole-genome sequencing confirmed no deletions in the spike multibasic cleavage domain (9).

A549-ACE2 cells were seeded at 10E4 cells per well. The next day, cells were infected with SARS-CoV-2 (USA-WA1/2020) at a multiplicity of infection of 0.05 in DMEM containing 2% FBS. After 2 hours of incubation at 37°C, the viral inoculum was removed, cells were washed with PBS, and 100 µL DMEM containing 10% FBS was added. Culture supernatants were harvested 24 hours post-infection, and the virus was inactivated by adding isopropanol to a final concentration of 80%, followed by a 10 min incubation at room temperature (12). Inactivated supernatants from cell culture with and without SARS-CoV-2 infection (SARS-CoV-2 infected, *n* = 2; SARS-CoV-2 uninfected, *n* = 2) were tested by tandem mass spectrometry as described above.

### Machine learning analysis

Data were exported from Excel to Python for machine learning (ML) analysis. Data preprocessing was performed using quantile scaling to output a normal distribution. To identify potential biomarkers for SARS-CoV-2 infection, ML analysis was first performed on the preprocessed data of the discovery cohort (LC/Q-TOF). The full data set was randomly divided into a training set (80% of samples) and a test set (20% of samples) to provide balance between the model learning effectively and reserving a separate subset for testing. The training set was used to build the machine learning models, and the test set was used to evaluate the test performance of the biomarker signature using the area under the receiver operating characteristic curve (AUC). Shapley additive explanations (SHAP) summary plots were generated for each machine learning model to highlight the top 20 features and their individual contribution, as is standard practice with this analysis to better understand the impact of each feature toward model prediction (13). Four models were used to assess the top 20 features of SARS-CoV-2 infection: two statistical models (Lasso and logistic regression) and two ML models (Random Forest [RF] and Light Gradient Boosting Model [LGBM]). The two traditional statistical models were used to investigate test performance of models assuming a linear relationship between features and outcome. In addition, the two machine learning models were investigated to draw on these models’ robustness for complex datasets and ability to capture nonlinear relationships, as described in more detail previously (3). Analyses were performed using Python 3.9.16, with LGBM v.3.1.0 for gradient-boosted decision trees and scikit-learn v.0.23.2 for RF. Stratified *k*-fold cross-validation and grid search were used for hyperparameter tuning. SHAP v.0.36.0 was utilized to compute feature importance.

The abovementioned method was then applied to the preprocessed validation cohort data (LC/MS-MS) to further evaluate test performance. In addition to the binary classification pipeline described above, an adaptation was made to investigate the use of four separate ML models (three neural networks [NNets 1, 2, and 3] and one support vector machine) for multiclass classification to predict infection status into four groups (SARS-CoV-2, influenza A, RSV, and negative) based on the same data set. The same proportions for training and testing were used. Neural networks were prepared on TensorFlow v.2.15.0.

### Statistical analysis

Descriptive data analysis was performed using the chi-squared and Mann-Whitney *U* tests for continuous variables, implemented in R v.4.0.2. The impact of potential confounders determined a priori*,* including age, sex, and machine learning model output, was investigated with multivariable analysis, as previously described (3).

## RESULTS

### Discovery cohort description

**Fig 1:**
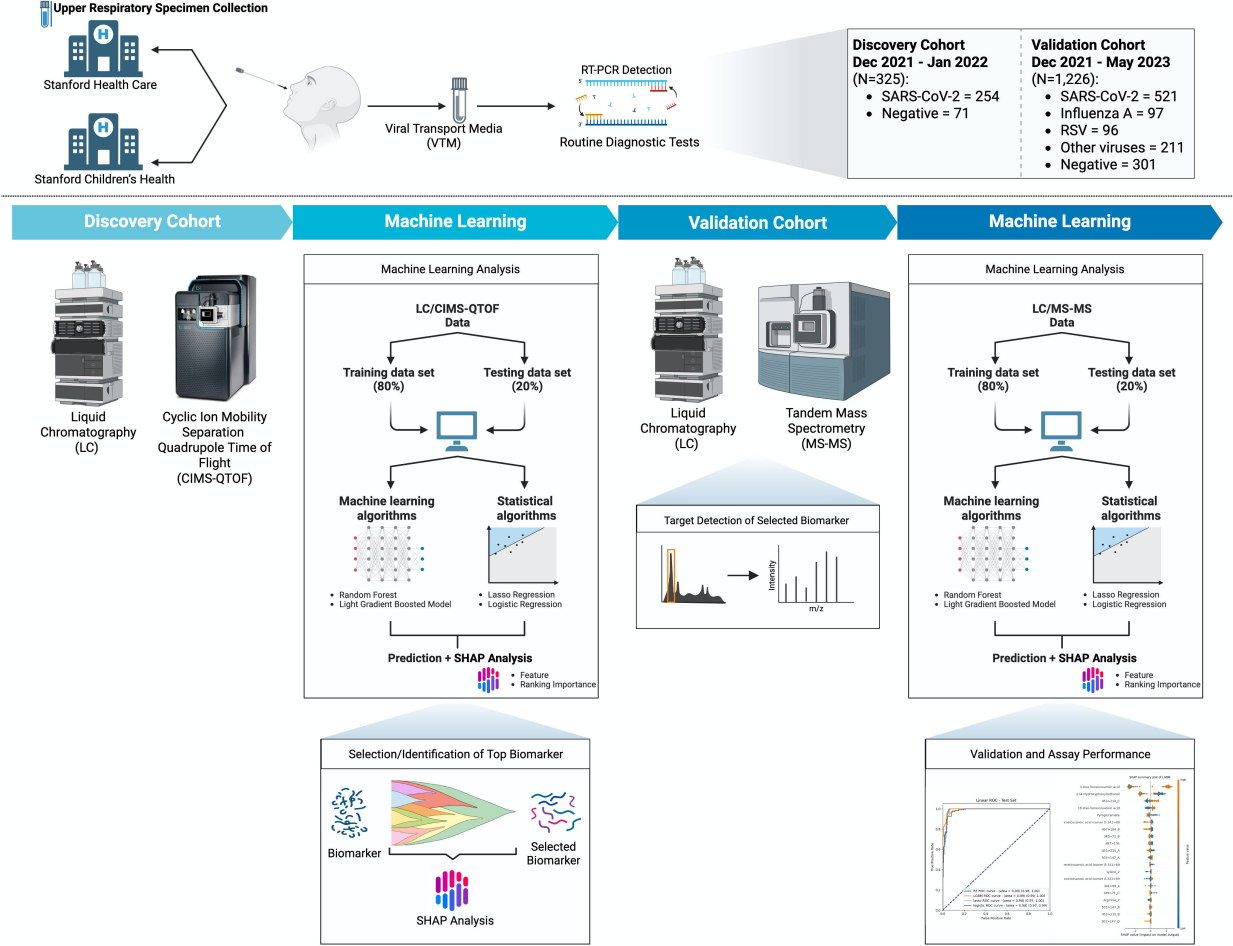
Respiratory virus metabolomics study flowchart. CIMS-Q-TOF, cyclic ion mobility separation quadrupole-time of flight; LC, liquid chromatography; LC-MS/MS, liquid chromatography-tandem mass spectrometry; RSV, respiratory syncytial virus; RT-PCR, reverse transcriptase polymerase chain reaction; SARS-CoV-2, severe acute respiratory syndrome coronavirus type 2; SHAP, Shapley additive explanations.

A total of 325 samples were tested in the discovery cohort (Fig. 1) (Table S1). All samples were successfully tested, and no sample was excluded from analysis. Of these, 254 were positive for SARS-CoV-2, and 71 were negative for SARS-CoV-2 by NAAT. Whole-genome sequences were obtained for 99.6% (253 out of 254), all of which were Omicron subvariants. Overall, the median age was 41 years (29–55 years), and 55.4% of the participants were female. Most samples were nasopharyngeal swabs (66.5%), followed by nasal (31.1%) and mid-turbinate swabs (2.5%) (Table S1).

### Test performance of the discovery cohort by LC/Q-TOF

The top model, LGBM, achieved an AUC of 1.00 (95% confidence interval [CI], not available) for classifying SARS-CoV-2 infection versus negative from respiratory swabs (Fig. S1). Other models also showed high performance, including RF with an AUC of 0.99 (95% CI, 0.97–1.00) and Lasso with an AUC of 0.99 (95% CI, 0.98–1.00). SHAP analysis based on the LGBM and RF models identified 341.3050 at 12.29 (written as *m*/*z* at retention time) as the top-ranking feature for classification performance of SARS-CoV-2 infection status (Fig. S2). This feature was associated with higher risk of SARS-CoV-2 infection. The top 20 compound signatures identified through SHAP analysis were subsequently incorporated into the targeted metabolomics approach for the validation cohort.

### Validation cohort description

A total of 1,226 samples were tested in the validation cohort (Table 1). All samples were successfully tested, and no sample was excluded from analysis. Of these, 521 were positive for SARS-CoV-2; 404 samples were positive for other respiratory viruses; and 301 were negative for the viruses above. Whole-genome sequences were obtained for 91.0% (474 out of 521), and all were Omicron subvariants (BA.1.X, *n* = 70; BA.2.X, *n* = 94; BA.5.X, *n* = 34; BQ.1.X, *n* = 168; XBB0.10, *n* = 9; XBB.1.X, *n* = 42; XBB.2.X, *n* = 4; other, *n* = 53). The validation cohort included 404 samples positive for other respiratory viruses (influenza A, *n* = 97; RSV, *n* = 96; hMPV, *n* = 50; AdV, *n* = 51; RV, *n* = 50; PIV-1, *n* = 11; PIV-2, *n* = 9; PIV-3, *n* = 22; and PIV-4, *n* = 18). Six cases of viral co-infection were also characterized, including two SARS-CoV-2/influenza A, three SARS-CoV-2/RSV, and one SARS-CoV-2/rhinovirus. The baseline demographic and clinical characteristics of the validation cohort are described in Table 1. The median age was 42 years (14–65 years), and 49.8% of the participants were female. All samples included were nasopharyngeal swabs.

**TABLE 1:** Clinical characteristics of the individuals included in the validation phase of the study

	Overall cohort (%)	SARS-CoV-2 (%)	Influenza A virus (%)	RSV (%)	Other^*a*^ (%)	Negative^*b*^ (%)
Number included	1,226	521	97	96	211	301
Median age (Q1–Q3)	42 (14–65)	44 (21–65)	23 (10–50)	33.5 (4–60.5)	39 (6–63)	53 (18–70)
Age (years), no. (%)						
<18	336 (27.4)	115 (22.0)	41 (42.3)	41 (42.7)	69 (32.7)	70 (23.3)
≥18	890 (72.6)	406 (77.8)	56 (57.7)	55 (57.3)	142 (67.3)	231 (76.7)
Sex, no. (%)						
Male	597 (48.7)	241 (46.3)	56 (57.7)	41 (42.7)	108 (51.2)	151 (50.2)
Female	629 (51.3)	280 (53.7)	41 (42.3)	55 (57.3)	103 (48.8)	150 (49.8)
Hospitalized						
Yes	851 (69.4)	406 (77.9)	47 (48.5)	43 (44.8)	91 (43.1)	235 (78.1)
No	375 (30.6)	115 (22.1)	50 (51.5)	53 (55.2)	120 (56.9)	66 (21.9)

^*a*^ Includes adenovirus, human metapneumovirus, rhinovirus, and parainfluenza virus.

^*b*^ Negative for all respiratory viruses.

### Test performance of the validation cohort by LC/MS-MS

**Fig 2:**
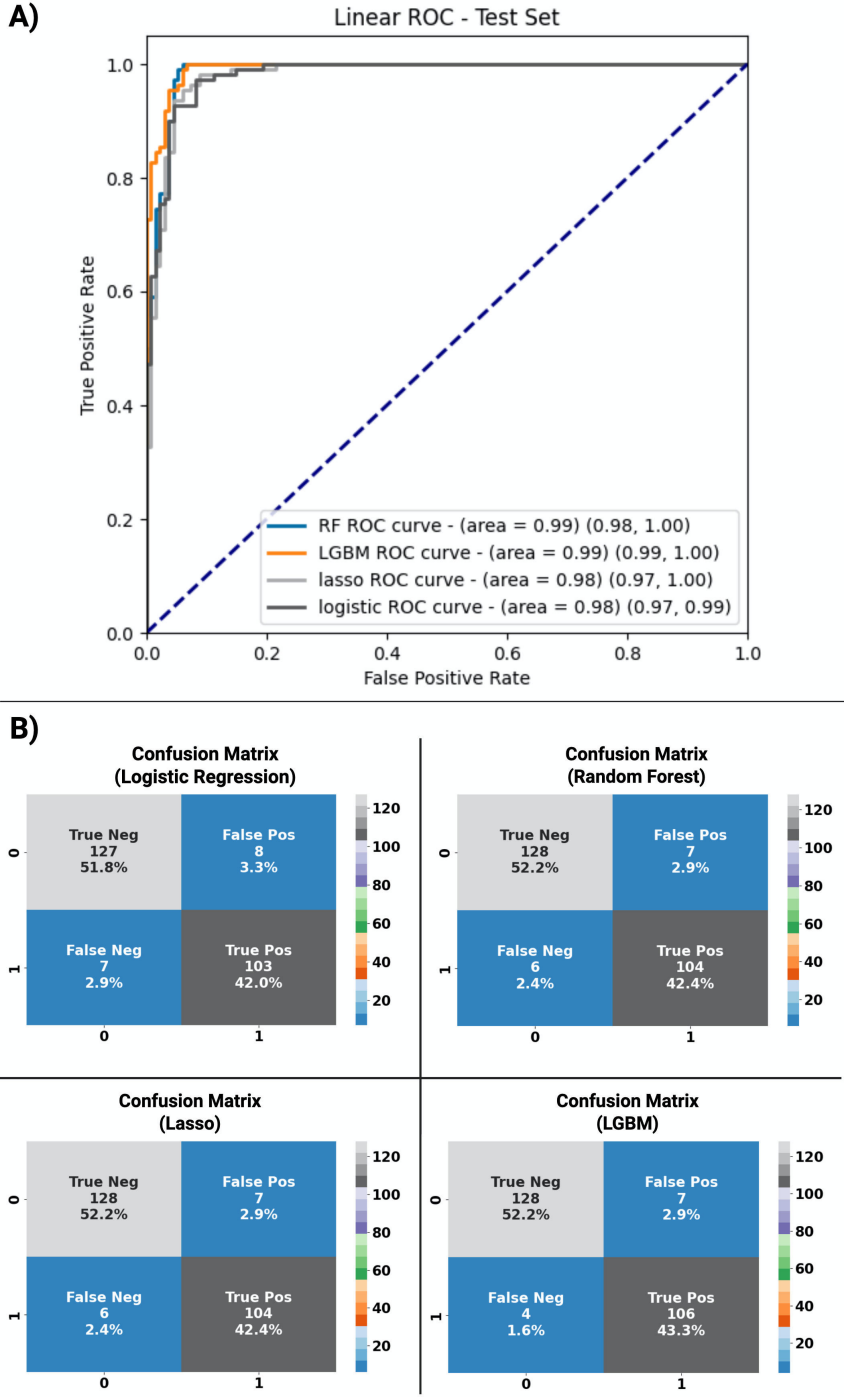
Receiver operating characteristic (ROC) curve analysis and confusion matrices of the validation cohort. (**A**) ROC for the classification of SARS-CoV-2 infection by each model with area under the receiver operating characteristic curve shown for each model with associated 95% CI in parentheses. Robust performance was observed across all models with the highest performance observed with LGBM. (**B**) Confusion matrices depicting data underpinning sensitivity and specificity calculations for each model.

**Fig 3:**
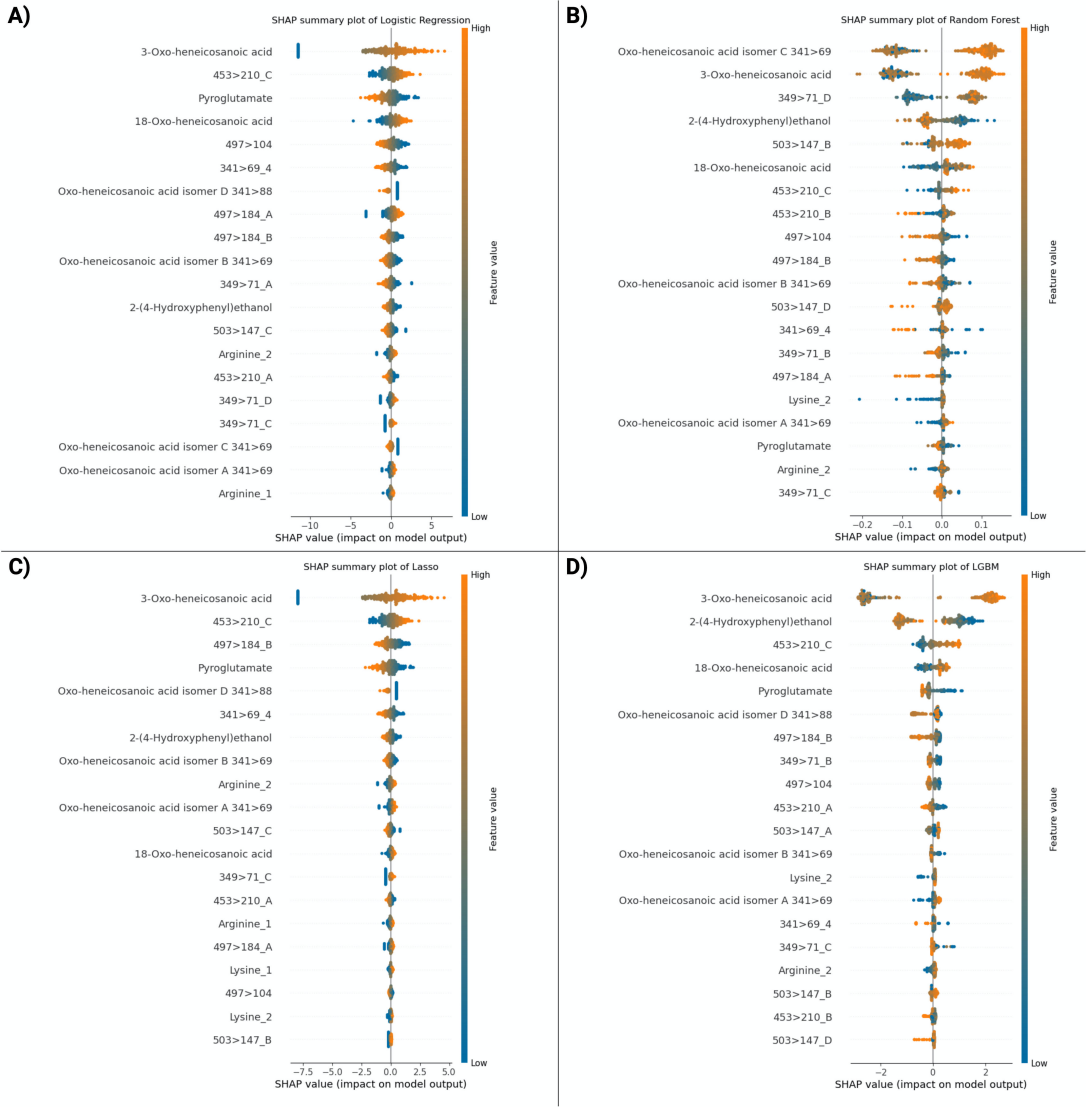
Shapley additive explanation (SHAP) analysis of the validation cohort. (**A–D**) Feature importance analysis by SHAP values for each model. The *y* axis represents the top 20 features of each model. The *x* axis represents the predicted SHAP output value scale. Positive SHAP values indicate model prediction of SARS-CoV-2 positivity, and negative SHAP values indicate model prediction of SARS-CoV-2 negativity. Features that are associated with higher risk of SARS-CoV-2 are presented in blue, and features associated with lower risk of SARS-CoV-2 are presented in orange. The top two features of the best-performing model LGBM, 3-oxo-heneicosanoic acid and 2-(4-hydroxyphenyl) ethanol, accounted for most of the test performance of the model as evidenced by the greater positive and negative SHAP values. The oxo- position could not be resolved for three features, listed as oxo-heneicosanoic acid isomers A, B, C, and D.

**Fig 4:**
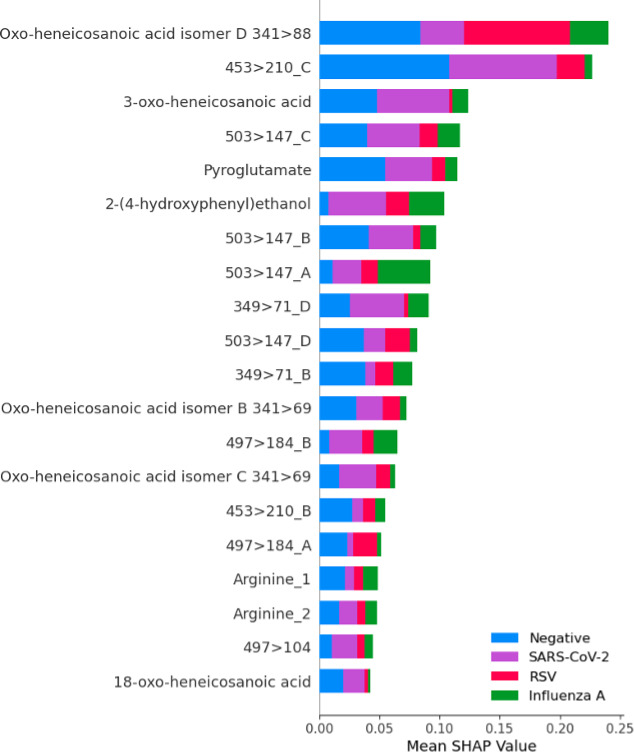
Multiclass analysis using Shapley additive explanation (SHAP) values. The *y* axis represents the top 20 features for the top-performing model, LGBM. The *x* axis represents the predicted SHAP output value scale. The relative importance of each feature is indicated by the magnitude of the mean SHAP value in the figure above. The mean SHAP value for each class is encoded as negative (blue), SARS-CoV-2 (purple), RSV (red), influenza A virus (green). The 3-oxo-henecosanoic acid compounds (main compound and related isomers) showed greater relative feature importance in SARS-CoV-2-positive, RSV-positive, and negative samples than for influenza A-positive samples.

All models achieved an AUC of 0.98 or greater for classifying SARS-CoV-2 infection versus negative when including the full data set with viral coinfections. The top-performing model was LGBM with an AUC of 0.99 (95% CI, 0.99–1.0), sensitivity of 0.96 (95% CI, 0.91–0.99), and specificity of 0.95 (95% CI, 0.90–0.97) (Table 2; Fig. 2). Feature ranking by SHAP analysis based on LGBM showed that two compounds accounted for most of the test performance such that biomarker identification efforts were focused on these compounds (Fig. 3). Compound 341 > 88.2 (written as precursor ion >fragment ion, which corresponds to compound 341.3050 at 12.29, written as m/z at retention time from the discovery set) was the top-ranking feature and was subsequently identified as 3-oxo-heneicosanoic acid (Fig. 3). More specifically, high concentrations of this compound showed the greatest impact on model performance and was the strongest predictor for SARS-CoV-2 infection in this data set. The second most important feature was 139 > 77, which was identified as 2-(4-hydroxyphenyl) ethanol when compared with commercially available material. High concentrations of this compound predicted the absence of SARS-CoV-2 infection. Median concentrations of the top 20 features in SARS-CoV-2-positive and SARS-CoV-2-negative samples are presented separately (Table S2). Exclusion of the six cases of viral co-infection showed similar overall model performance, with an AUC of 1 (95% CI, 0.99–1.00) for the top-performing model, LGBM, for the main analysis of classification of SARS-CoV-2-positive versus SARS-CoV-2-negative samples. A multivariable machine learning model incorporating a priori defined potential confounders (age and sex) demonstrated that only the outcome of the machine learning model was significantly associated with SARS-CoV-2 infection status (Table S3). The same biomarker signature originally derived for SARS-CoV-2 was then applied to the same dataset, including viral coinfection but using multiclass analysis to include separate categories for SARS-CoV-2-positive, influenza A-positive, RSV-positive, and negative samples for any of these three viruses. This signature showed high performance for the classification of influenza A with an AUC of 0.97 (95% CI, 0.94–0.99) and RSV with an AUC of 0.99 (95% CI, 0.97–1.00). The relative importance of each feature in the multiclass model is illustrated in Fig. 4. The 3-oxo-henecosanoic acid compounds (main compound and related isomers B, C, and D) showed a trend toward greater relative feature importance for SARS-CoV-2 and RSV, compared to influenza A (Fig. 4).

**TABLE 2:** Area under the curve, sensitivity, and specificity of the four models investigated in the study for classification of SARS-CoV-2-positive versus SARS-CoV-2-negative samples

	LGBM	RF	Lasso	Logistic regression
AUC (95% CI)	0.99 (0.99–1)	0.99 (0.98–1)	0.98 (0.97–1)	0.98 (0.97–0.99)
Sensitivity (95% CI)	0.96 (0.91–0.99)	0.95 (0.89–0.97)	0.95 (0.89–0.97)	0.94 (0.87–0.97)
Specificity (95% CI)	0.95 (0.90–0.97)	0.95 (0.90–0.97)	0.95 (0.90–0.97)	0.94 (0.89–0.97)

### Biomarker feature identification

The top-ranking SARS-CoV-2 feature was identified as a compound with a *m*/*z* ratio of 341.3050, corresponding to oxo-heneicosanoic acid (C21H40O3), though the position of the oxo- group can vary from C2 to C20. Tandem mass spectrometry demonstrated major fragments at *m*/*z* 69.1 and 88.1 in the positive cases, suggesting the presence of multiple oxo-isomers. Based on machine learning analysis, the SRM pair 341.3 > 88.1 was identified as a key marker for SARS-CoV-2 detection, prompting further focus on this compound. The fragment ion at *m*/*z* 88.1 suggested that the oxo group is located at the C3 position, forming the fragment O = CCH2CO_2_H + H + , which was later confirmed using synthetic 3-oxo-heneicosanoic acid, CH_3_(CH_2_)17(CO)CH_2_CO_2_H. Compared with the candidate biomarker, synthetic 3-oxo-heneicosanoic acid demonstrated identical SRM pair and retention times when examined using an orthogonal chromatography column. The SARS-CoV-2-infected cell supernatant showed an approximately 100-fold greater concentration of 3-oxo-heneicosanoic acid compared to the uninfected cell supernatant (Fig. S4). Additionally, the chromatogram of the synthetic 3-oxo-heneicosanoic acid matched those of SARS-CoV-2-positive patient samples and supernatant from SARS-CoV-2-infected cells.

The second-ranked SARS-CoV-2 biomarker had a *m*/*z* ratio of 139.073. Tandem mass spectrometry (LC-MS/MS) revealed a major fragment ion at *m*/z 77.04, which is consistent with the benzenium ion in either 4-ethoxyphenol or 2-(4-hydroxyphenyl) ethanol. The 139.073 > 77.04 peak was present by LC/MS-MS in the uninfected cell supernatant and absent in the infected cell supernatant. Compared with the top candidate biomarker, standard 4-ethoxyphenol produced no signal for the SRM pair 139.1 > 77. However, 2-(4-hydroxyphenyl) ethanol displayed an identical SRM pair of 139.1 > 77 and a nearly identical retention time, confirming the biomarker as 2-(4-hydroxyphenyl) ethanol. This compound likely presents the hydroxyl group in the para position relative to the ethanol group, and clinical samples may contain a mixture of para-, meta-, and ortho-isomers.

## DISCUSSION

In this study, we performed comprehensive metabolic profiling of upper respiratory samples combined with machine learning analysis to identify a top biomarker signature as a potential discriminator between SARS-CoV-2-infected and SARS-CoV-2-uninfected individuals. Through a discovery cohort of 325 samples from adult and pediatric inpatients and outpatients from a single health center, we identified a 20-feature biomarker signature by LC/Q-TOF with the potential to classify individuals with and without SARS-CoV-2 infection directly from upper respiratory swab samples. We further investigated the test performance of this signature in an independent cohort of 1,226 samples and demonstrated >0.95 AUC, sensitivity, and specificity for the top ML model, LGBM. Model performance was driven by two main compounds of interest, which were identified as 3-oxo-heneicosanoic acid and 2-(4-hydroxyphenyl) ethanol. Importantly, although the signature was derived from a discovery cohort selected for SARS-CoV-2 positive versus negative, it maintained a high level of performance when extended to the differentiation of influenza A virus and RSV, supporting the potential use of this signature for other respiratory viruses.

The biomarker signature, which includes 20 distinct features, highlighted 3-oxo-heneicosanoic acid and 2-(4-hydroxyphenyl) ethanol as the top two compounds contributing to the robust classification performance of this method. The first compound, 3-oxo-heneicosanoic acid, is a 21-carbon, long-chain saturated oxo fatty acid. That such a compound might differentiate SARS-CoV-2 from other respiratory viruses was not entirely surprising, given that SARS-CoV-2-infected individuals have been shown to have altered lipid metabolism (14, 15). Nevertheless, the biological role of 3-oxo-heneicosanoic acid is not well understood, and odd-chain fatty acids are uncommon in human metabolism. Interestingly, individuals with COVID-19 have elevated levels of odd-chain fatty acids in their sebum lipidome compared to healthy controls (16). The important role of the respiratory microbiota in SARS-CoV-2 infection also raised concerns that the 3-oxo-heneicosanoic acid detected in these respiratory samples may be derived from bacteria in the upper respiratory tract (17–19). However, the presence of 3-oxo-heneicosanoic acid in the supernatant of human cell culture, at higher levels in the supernatant of SARS-CoV-2-infected cells but present in the supernatant of uninfected cells as well, strongly supports an endogenous source.

The compound 3-oxo-heneicosanoic acid can be formed from disrupted β-oxidation or cells under oxidative stress (20, 21). Mitochondria and peroxisomes are the major organelles for the metabolism and regulation of long-chain fatty acids and very long-chain fatty acids and are the locations within the cell where β-oxidation takes place (22, 23). SARS-CoV-2 has been shown to interfere with mitochondrial and peroxisomal functions (24, 25). The increase in 3-oxo-heneicosanoic acid we observed may result from SARS-CoV-2-induced dysregulation of β-oxidation pathways and alterations in mitochondrial and peroxisomal functions. However, no direct evidence currently supports this hypothesis, and the origin of this odd-chain fatty acid remains unknown. Further investigation is required to elucidate the mechanisms underlying the accumulation of 3-oxo-heneicosanoic acid in SARS-CoV-2-infected cells and its potential role in COVID-19 pathophysiology.

The second most important compound identified for differentiating SARS-CoV-2 infection from other respiratory virus infections was 2-(4-hydroxyphenyl) ethanol, also known as tyrosol. Endogenous tyrosol is a metabolite of the amino acid tyrosine, and this compound is also found in various dietary sources, such as olive oil (26). Tyrosol possesses relatively weak antioxidant properties, though its stability and capacity for intracellular accumulation render tyrosol a durable antioxidant, especially during inflammation (27, 28). Tyrosol can inhibit H_2_O_2_-induced cell death and regulate key signal transduction pathways (29). Additionally, tyrosol and its metabolites have been shown to reduce the level of reactive oxygen species (ROS) and prevent the activation of NF-κB in tumor necrosis factor alpha (TNF-α)-treated human endothelial cells (30). ROS production and TNF-α are the key players in the host antiviral immune response, and excessive activation of these responses can lead to tissue damage (31, 32). Hydroxytyrosol, a derivative of tyrosol, can reduce the expression of the SARS-CoV-2 papain-like protease and apoptosis in infected epithelial cells (33). Additional experiments will be required to investigate how SARS-CoV-2 infection results in decreased levels of 2-(4-hydroxyphenyl) ethanol.

The performance of this biomarker signature suggests potential utility as a diagnostic test for respiratory virus infection, and though mass spectrometry is complex, the method described in this study has several advantages over respiratory virus NAAT. The LC/MS-MS method requires limited preanalytical processing (the addition of isopropanol followed by centrifugation), and analysis on the instrument requires less than 5 min per sample. Mass spectrometry is also inexpensive on a per-sample basis (reagent cost: ~10 cents per sample) compared to NAAT (reagent cost: ~10 USD to >150 USD, depending on panel size). In addition, an LC/MS-MS instrument is similar in price to an automated NAAT system, such as the Hologic Panther. Notably, SHAP feature importance analysis demonstrated that only a few compounds account for most of the test performance. This supports adaptation of this diagnostic approach to a simpler modality that could be performed in the near-care setting, including existing portable mass spectrometers.

The testing approach presented in this study is valuable as it draws from minimally invasive sample collection and benefits from two large sets of samples separated into discovery and validation sets. The data presented directly extend our understanding of the key metabolites that may differentiate SARS-CoV-2-infected from SARS-CoV-2-uninfected individuals and pave the way for an alternative diagnostic approach for viral identification that may complement current testing strategies. Furthermore, the analysis spans a comprehensive range of two statistical and two machine learning models, which demonstrated the reproducibility of findings across models and supported the robustness of the approach. Nonetheless, several limitations should be highlighted. First, these samples were collected retrospectively after a variable period of freezer storage and without controlling for several variables that may confound results that a prospective study would have been better suited to address. This includes timing since symptom onset, disease severity, vaccination status, and impact of therapeutics. Nonetheless, based on the demographic data collected and due to the large cohort studied, this impact may have been lessened through the distribution of these factors between the groups. Second, these data were generated from Stanford Health Care, a single health care system, and further work is required to assess generalizability of this diagnostic approach. Third, the positive sample set included only Omicron subvariants, and was selected based on successful sequencing results for the SARS-CoV-2-positive samples, resulting in a limited distribution of viral burden as estimated by cycle threshold values. Future work should incorporate additional SARS-CoV-2 variants, as well as a wider range of cycle threshold values to evaluate potential dose-response relationships and to confirm signature performance in samples with a low viral burden. More comprehensive analysis of additional respiratory sample types, including lower respiratory tract samples, may also be valuable. Fourth, our data set did not incorporate bacterial coinfection data, which may have confounded results. Last, due to the exploratory nature of this work, the mechanistic explanations for the metabolomic changes detected in SARS-CoV-2 samples remain largely speculative. Additional studies are needed to investigate these metabolic pathways in depth and determine how their changes contribute to the differentiation between SARS-CoV-2-positive and SARS-CoV-2-negative samples.

In summary, we identified a biomarker signature differentiating SARS-CoV-2-positive versus SARS-CoV-2-negative samples directly from upper respiratory samples. We subsequently demonstrated the performance of this signature based on an independent validation cohort by targeted mass spectrometry combined with machine learning analysis. This work suggests that mass spectrometry-based infectious disease diagnostic testing has clinical potential and that these metabolomic features may reveal novel host-pathogen interactions and therapeutic targets. In addition to SARS-CoV-2, we believe that applying a similar approach to prospective, multisite cohorts of patients with other infectious diseases can greatly extend our understanding of the metabolic pathways involved in the host response to infection.

## Data Availability

The code generated during this study is available on request.

## SUPPLEMENTAL MATERIAL

Supplemental material jcm.02042-24-s0001.pdf Tables S1 to S3, Fig. S1 to S4, and Appendix 1 (synthesis of 3-oxohenicosanoic acid).

ASM does not own the copyrights to Supplemental Material that may be linked to, or accessed through, an article. The authors have granted ASM a non-exclusive, world-wide license to publish the Supplemental Material files. Please contact the corresponding author directly for reuse.
