# Supplementary material for: Comprehensive metabolomics combined with machine learning for the identification of SARS-CoV-2 and other viruses directly from upper respiratory samples
Source: J Clin Microbiol. 2025 Oct 9;63(11):e02042-24. doi: 10.1128/jcm.02042-24 (PMC12607797; doi:10.1128/jcm.02042-24)
Supplement: Supplemental material — Tables S1 to S3, Fig. S1 to S4, and Appendix 1 (synthesis of 3-oxohenicosanoic acid). [file jcm.02042-24-s0001.pdf]

Supplemental Material for

Comprehensive metabolomics combined with machine learning for the identification of SARS-CoV-2 and other viruses directly from upper respiratory samples

Catherine A. Hogan, Anthony T. Le, Afraz Khan, LingHui David Su, ChunHong Huang, Malaya K. Sahoo, Chieh-Wen Lo, Marwah Karim, Karin Ann Stein, Shirit Einav, Tina M. Cowan, Benjamin A. Pinsky

Correspondence to: bpinsky@stanford.edu

This file includes:

Supplemental Tables 1 to 3
Supplemental Figures 1 to 4

Supplemental materials

Appendix 1. Synthesis of 3-oxohenicosanoic acid.

Nonadecanoic acid (250 mg, 0.84 mmol) was suspended in dichloromethane (4 mL) under argon atmosphere. Meldrum's acid (130 mg, 0.92 mmol) was added, followed by 1-ethyl-3-(3-dimethylaminopropyl carbodiimidehydrochloride (EDCI, 208 mg, 1.09 mmol) and dimethylaminopyridine (DMAP, 10 mg), and the reaction was stirred for 3 hours at ambient temperature. The reaction was diluted with ethyl acetate (50 mL), washed with water (10 mL) and brine (10 mL), dried and concentrated to a yellow oil. The product was purified by column chromatography (10g SiO_2 column, eluent 20-70% ethyl acetate/hexane) to provide 2,2-dimethyl-5-nonadecanoyl-1,3-dioxane-4,6-dione (Compound 1).

Compound 1 (100 mg, 0.24 mmol) was dissolved in toluene (1mL). Benzyl alcohol (0.040 mL, 0.35 mmol) was added, and the reaction was heated at 100°C overnight. The reaction was concentrated and chromatographed (10g SiO_2 column, 1-15% ethyl acetate/hexane) to provide benzyl 3-oxohenicosanoate (Compound 2).

Compound 2 (41 mg, 0.10 mmol) was dissolved in ethanol (5 mL). 10% palladium on carbon (Pd/C, 8 mg) was added and the reaction was stirred under hydrogen atmosphere for 30 minutes. The catalyst was removed by filtration and the reaction was concentrated. The product was purified by column chromatography (10g SiO_2 column, 1-10% MeOH/CH_2Cl_2) to provide 3-oxohenicosanoic acid (Compound 3).

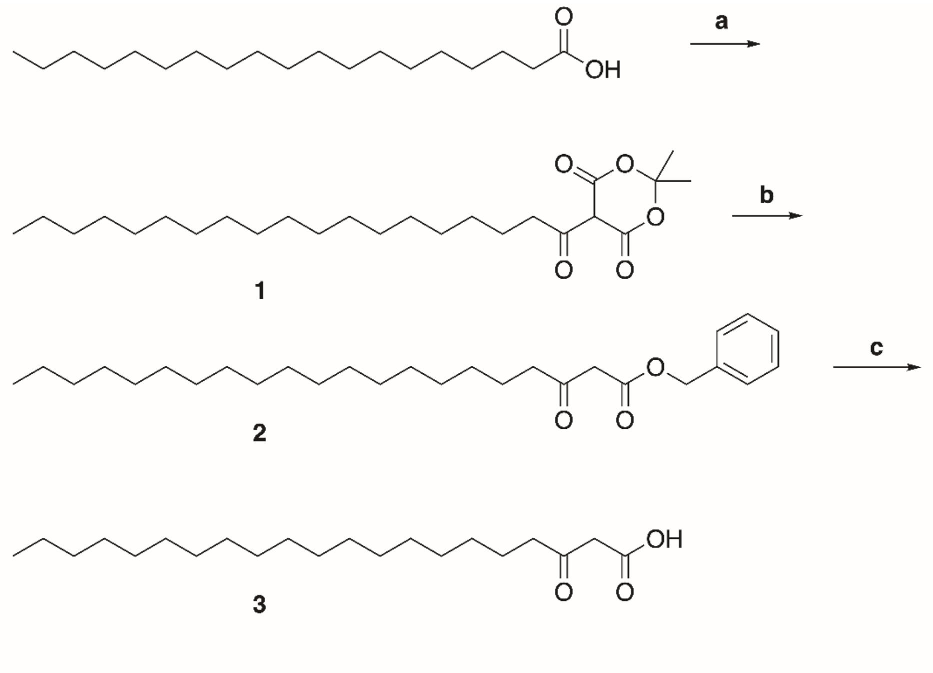

Scheme 1: a. Meldrum's acid, EDCI, DMAP, CH_2Cl_2; b. BnOH, toluene; c. H_2, Pd/C, EtOH

Supplemental Table 1. Demographic, clinical and laboratory characteristics of the individuals included in the discovery cohort.

		**Overall cohort**	**SARS-CoV-2 (%)**	**Negative for any respiratory virus (%)**
**Number included**		325	254 (78.2)	71 (21.8)
**Median age (Q1-Q3)**		41 (29-55)	45 (29.3-59)	36 (29-45.5)
**Age (No. [%])**	<18yo	22 (6.8)	16 (6.3)	6 (8.5)
	≥18yo	303 (93.2)	238 (93.7)	65 (91.5)
**Sex (No. [%])**	Male	145 (44.6)	125 (49.2)	20 (28.2)
	Female	180 (55.4)	129 (50.8)	51 (71.8)
**Hospitalized**	Yes	158 (48.6)	152 (59.8)	65 (91.5)
	No	167 (51.4)	102 (40.2)	6 (8.5)
**Sample type**	Nasal	101 (31.1)	43 (16.9)	58 (81.7)
	Mid-turbinate	8 (2.5)	3 (1.2)	5 (7.0)
	Nasopharyngeal	216 (66.5)	208 (81.9)	8 (11.3)

No.: number; SARS-CoV-2: severe acute respiratory syndrome coronavirus type 2; yo: years old.

Supplemental Table 2. Median peak areas of the top 20 features in SARS-CoV-2 positive and negative samples.

Compound	Median peak value (IQR)		Positive : Negative ratio	p-value[a]
	SARS-CoV-2 Positive	SARS-CoV-2 Negative		
3-oxo-heneicosanoic acid 341>88	318034.5 (136021.0)	98269.0 (96921.0)	3.24	1.5e-37
2-(4-hydroxyphenyl) ethanol 139>77	7650.0 (6767.25)	37286.0 (26284.5)	0.21	2.0e-36
453>210	84584.5 (12035.0)	53821.0 (25898.0)	1.57	5.2e-27
18-oxo-heneicosanoic acid 341>69	320123.5 (103422.0)	312490.0 (164323.0)	1.02	0.8
Pyroglutamate	867069.0 (90580.5)	898296.0 (140567.5)	0.97	0.001
Oxo-heneicosanoic acid isomer 341>88_A	16042.0 (23598.5)	18103.0 (27932.5)	0.89	0.02
497>184_B	43321328.0 (31860368.5)	63102304.0 (26140832.0)	0.69	1.4e-11
349>71_B	562384.0 (122397.0)	620027.0 (171599.5)	0.91	0.001
497>104	42905518.0 (29432349.0)	59620980.0 (26336732.0)	0.72	9.9e-11
453>210_A	334218.5 (317467.75)	378983.0 (334887.0)	0.88	0.6
503>147_A	83184.0 (29078.5)	55278.0 (39733.5)	1.50	2.2e-12
Oxo-heneicosanoic acid isomer 341>69_C	278584.0 (60247.5)	307143.0 (98604.5)	0.91	3.9e-05
Lysine_B	230907.5 (195299.5)	117630.0 (144596.5)	1.96	1.3e-09
Oxo-heneicosanoic acid isomer 341>69	79853.5 (62068.0)	74860.0 (31154.5)	1.07	0.06
341>69_D	118960.0 (58237.75)	122644.0 (61320.5)	0.97	0.2
349>71_C	15581.5 (22711.75)	40994.0 (53483.0)	0.38	1.2e-15
Arginine_B	992296.5 (996260.75)	1142086.0 (906991.5)	0.87	0.05

503>147_B	313067.5 (141519.5)	131933.0 (140795.0)	2.37	3.3e-22
453>210_B	11112.5 (10381.0)	13185.0 (14738.0)	0.84	0.09
503>147_D	41678.5 (14457.75)	24821.0 (31428.5)	1.68	6.6e-10

[a]Mann-Whitney *U* test

All values represent measurements of area under the peak.

SARS-CoV-2: severe acute respiratory syndrome coronavirus type 2.

Supplemental Table 3. Multivariable linear regression model for SARS-CoV-2 status prediction adjusted for age, sex, and LGBM machine learning output. Only the LGBM output was observed to be significantly associated with SARS-CoV-2 infection status prediction.

	Coefficient	Standard error	P-value	95% CI
**Age**	0.1631	0.264	0.5	-0.35, 0.68
**Sex**	0.69	0.58	0.2	-0.44, 1.8
**LGBM output**	7.70	1.06	<0.001	5.63, 9.77

CI: confidence interval; LGBM: Light Gradient Boosted Model.

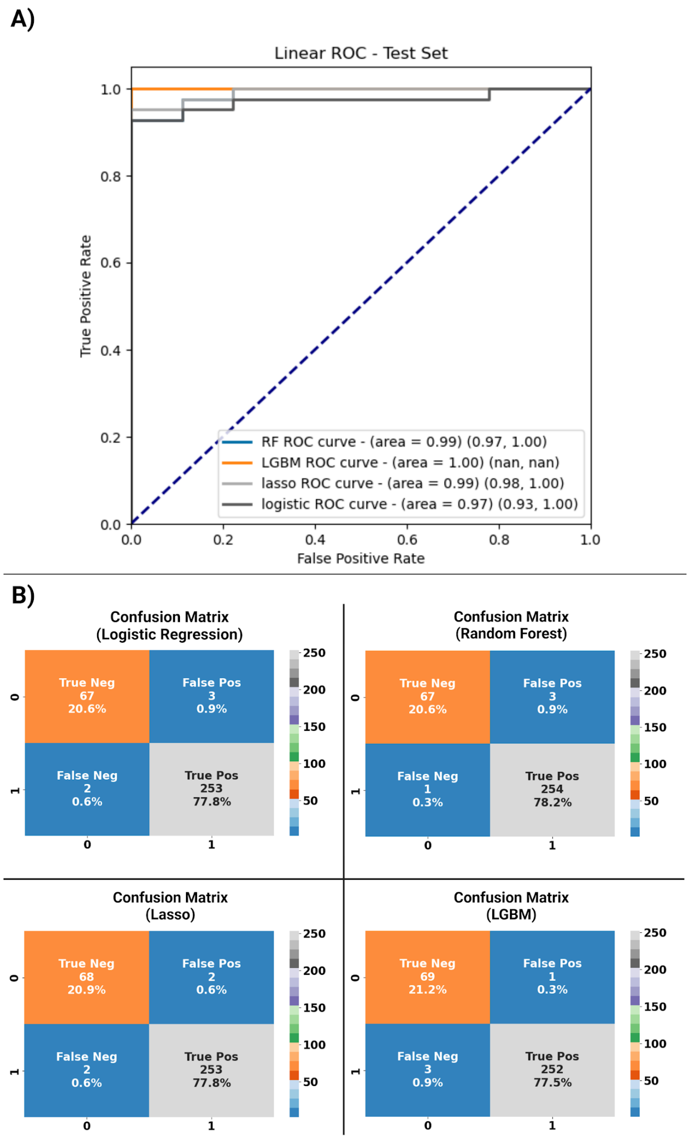

Supplemental Figure 1. Receiver Operating Characteristic Curve (ROC) Analysis and Confusion Matrices of the Discovery Cohort. A) ROC for the classification of SARS-CoV-2 infection by each model with area under the receiver operating characteristic curve (AUC) shown for each model with associated 95% confidence interval in brackets. Robust performance was observed across all models; however, the LGBM model showed slightly better performance. B) Confusion matrices depicting data underpinning sensitivity and specificity calculations for each model.

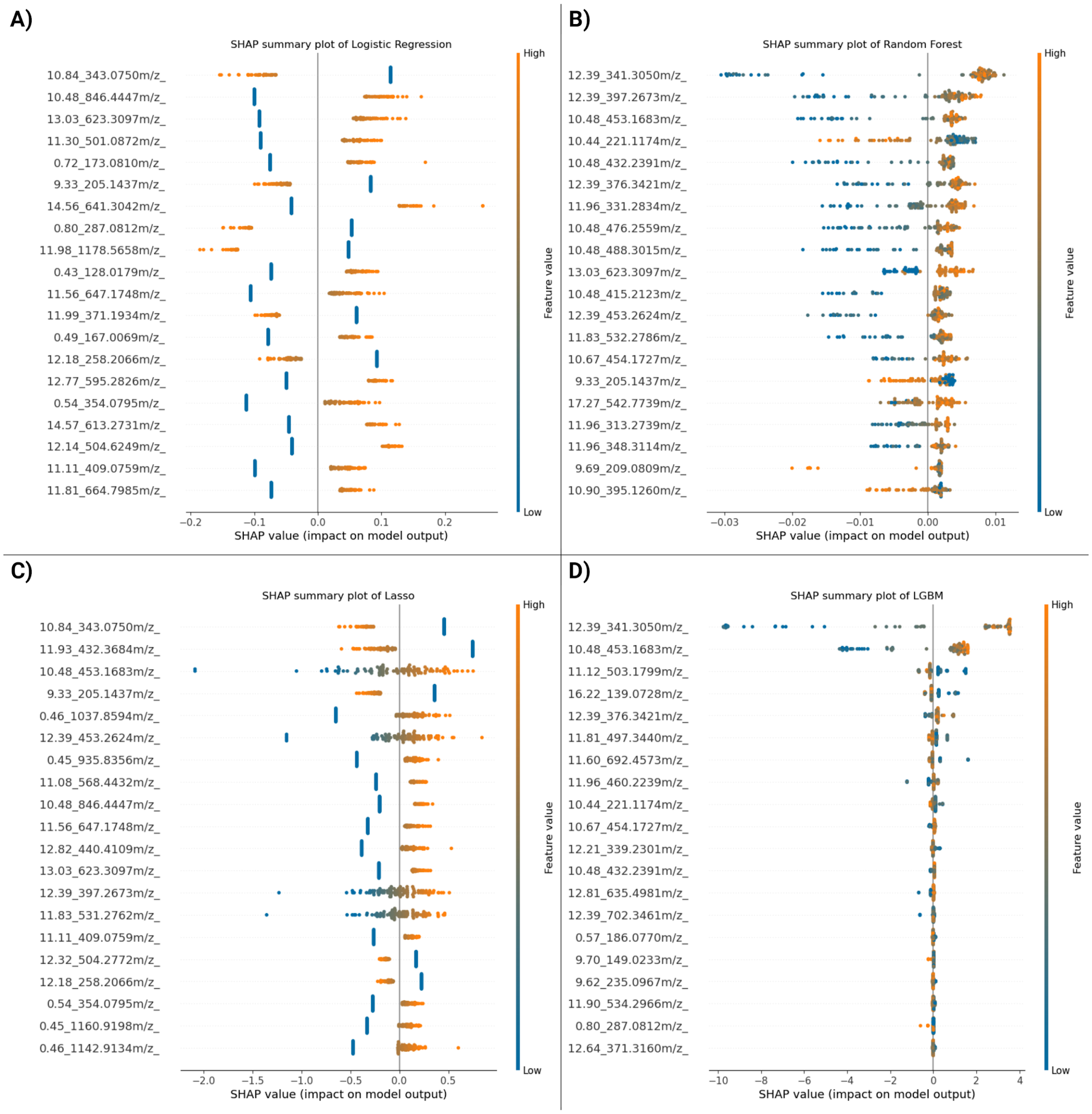

Supplemental Figure 2. SHapley Additive exPlanation (SHAP) Analysis of the Discovery Cohort. A-D) Feature importance analysis by SHAP values for each model. The y axis represents the top 20 features of each model. The x axis represents the predicted SHAP output value scale. Features that are associated with higher risk of SARS-CoV-2 are presented in blue, and features associated with lower risk of SARS-CoV-2 are presented in orange.

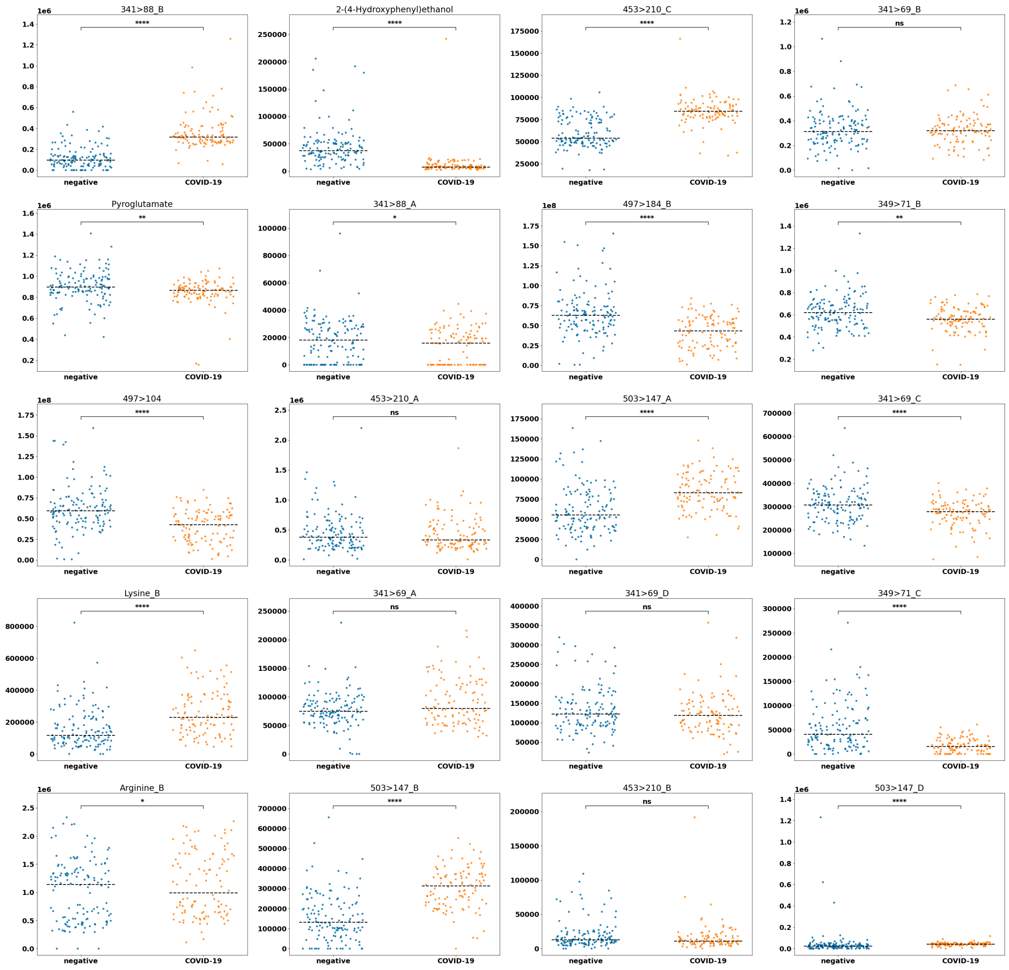

Supplemental Figure 3. Scatter Plots of the Top 20 Features Identified in the Validation Cohort by the Top Performing Model (LGBM). These features are shown for both SARS-CoV-2 negative (blue) and SARS-CoV-2 positive (orange) respiratory swabs. All y-axis values represent measurements of area under the peak. Statistical analysis was performed based on Mann-Whitney testing. ns: not significant (p>0.05). Negative group refers to samples tested negative for SARS-CoV-2.

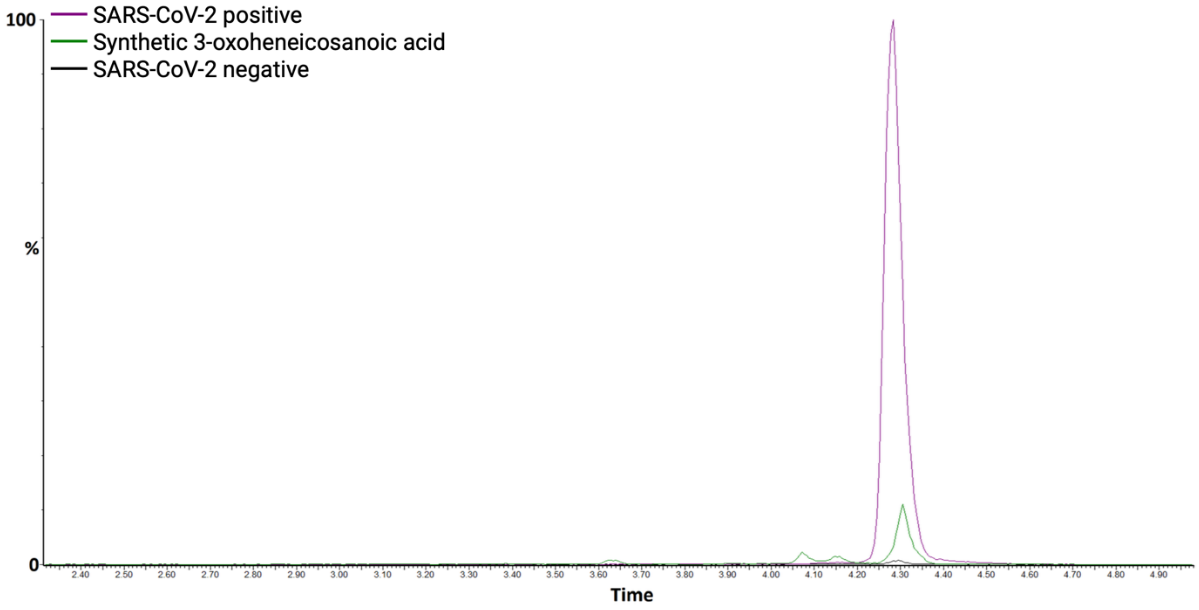

Supplemental Figure 4. Mass spectrometry chromatograms of the infected (purple) and uninfected (black) cell supernatants, and the synthetic 3-oxohenicosanoic acid (green). Based on peak area, the infected cell supernatant showed an approximately 100-fold higher concentration of 3-oxohenicosanoic acid compared to uninfected cell supernatant.
